# The functional mechanisms of synchronizing royal jelly consumption and physical activity on rat with multiple sclerosis-like behaviors hallmarks based on bioinformatics analysis, and experimental survey

**DOI:** 10.1186/s12868-022-00720-0

**Published:** 2022-06-08

**Authors:** Maryam Lohrasbi, Farzaneh Taghian, Khosro Jalali Dehkordi, Seyed Ali Hosseini

**Affiliations:** 1 Department of Sports Physiology, Faculty of Sports Sciences, Isfahan (Khorasgan) Branch, Islamic Azad University, Isfahan, Iran; 2 Department of Sport Physiology, Marvdasht Branch, Islamic Azad University, Marvdasht, Iran

**Keywords:** Multiple sclerosis, microRNAs, Hub genes, Royal Jelly, Physical activity, Biomarkers

## Abstract

**Background:**

Natural nutrition and physical training have been defined as non-pharmacochemical complementary and alternative medicines to prevent and treat various pathogenesis. Royal jelly possesses various pharmacological properties and is an effective therapeutic supplement for halting neurodegeneration. Multiple sclerosis is a prevalent neurodegenerative disorder that manifests as a progressive neurological condition. Inflammation, hypoxia, and oxidative stress have been identified as significant hallmarks of multiple sclerosis pathology.

**Results:**

In the present study, based on artificial intelligence and bioinformatics algorithms, we marked hub genes, molecular signaling pathways, and molecular regulators such as non-coding RNAs involved in multiple sclerosis. Also, microRNAs as regulators can affect gene expression in many processes. Numerous pathomechanisms, including immunodeficiency, hypoxia, oxidative stress, neuroinflammation, and mitochondrial dysfunction, can play a significant role in the MSc pathogenesis that results in demyelination. Furthermore, we computed the binding affinity of bioactive compounds presented in Royal Jelly on macromolecules surfaces. Also, we predicted the alignment score of bioactive compounds over the pharmacophore model of candidate protein as a novel therapeutic approach. Based on the q-RT-PCR analysis, the expression of the *Dnajb1/Dnajb1/Foxp1/Tnfsf14* and *Hspa4* networks as well as miR-34a-5p and miR155-3p were regulated by the interaction of exercise training and 100 mg/kg Royal Jelly (ET-100RJ). Interestingly, characteristics, motor function, a proinflammatory cytokine, and demyelination were ameliorated by ET-100RJ.

**Discussion:**

Here, we indicated that interaction between exercise training and 100 mg/kg Royal jelly had a more effect on regulating the microRNA profiles and hub genes in rats with Multiple sclerosis.

## Background

Neurodegenerative diseases are progressive conditions that occur through impairment in the function or structure of neurons in the peripheral nervous system or brain [1]. Moreover, This condition affects millions of individuals worldwide, and progressive degenerative diseases can influence body activities and functions such as breathing, moving, talking, and balance. For example, multiple sclerosis (MSc) is the prevalent neurodegenerative disorder caused by increasing neuroinflammation, which stimulates the immune cells and destroys neurons, axons, and myelin-producing oligodendrocytes [2].

Although several significant factors, including genetics, epigenetics, and environment, are associated with multiple sclerosis [3, 4], the pinpoint mechanism of MSc is not yet comprehended. However, several pathomechanisms such as immunodeficiency, hypoxia, oxidative stress, neuroinflammation, and mitochondrial dysfunction have been strongly involved in the MSc pathogenesis that leads to demyelination. Since increasing inflammation, hypoxia, and oxidative stress as dominant hallmarks are identified in multiple sclerosis pathology. Therefore, myelin Oligodendrocyte Glycoprotein (MOG) is widely applied as a model for demyelination. The dominant mechanism of MOG’s function is involved in inducing inflammation and oxidative stress, which leads to demyelination in the nervous system [5]. On the other hand, the artificial intelligence survey and protein–protein interaction network of data’s multiple sclerosis highlighted many principal hub genes involved in multiple sclerosis incidence and marked candidate proteins for the design and discovery of the suitable drug.

Hence, based on previous evidence around multiple sclerosis pathogenesis, the Heat Shock Protein Families such as HSP40 and HSP70 are defined as potentially protein families involved in the demyelination process [6]. Member B1 and B2 from the heat shock protein family with 40 kDa (HSP40) are termed DNAJB1and DNAJB2. Furthermore, HSPA4 from the heat shock protein family (70 kDa) is associated with demyelination in the peripheral nervous system and multiple sclerosis incidence [6]. Moreover, large-scale genome-wide association studies (GWAS) showed forkhead box protein P1 loci (foxp1) as a susceptible genomic location in multiple sclerosis have found differential expression patterns [7]. In addition, one of the crucial molecules involved in proliferation, differentiation, maturation, activation, and migration of the central nervous system is the tumor necrosis factor superfamily (TNFSF) [8]. Also, the clinical study revealed a strong association between autoimmune disorders such as multiple sclerosis and TNFSF [8].

In addition to suscept genes in multiple sclerosis pathogenesis, other agents such as environmental factors, epigenetic factors, and regulatory factors are associated with multiple sclerosis pathogenesis [3, 4, 9]. The genome’s non-coding sequences are usually involved in the post-transcriptional regulation of genes and gene expression. The microRNAs usually bind to the 3´UTR of coding genes and lead to inhibition or suppression of target genes. On the other hand, several studies suggested that the binding of microRNAs in 5´UTR of genes could consider the positive regulation of gene expression, leading to the induction of transcription [10].

Notably, there has been no practical and effective treatment strategy to cure or halt the development of MSc recently, and management of MSc remains complex. Therefore, numerous attempts have been conducted to understand the molecular mechanisms responsible for MSc to progress new therapeutic approaches for this condition [11, 12].

As unique and exceptional food for bee queen larvae nutrition, Royal jelly is secreted from the hypopharyngeal glands of the worker bees [13, 14]. The Royal jelly with complex and variable bioactive compounds relevant to seasonal and geographical area conditions is influenced by growth, development, reproduction, and lifespan [15]. Although the content of compounds is variable, Royal jelly includes proteins, lipids such as free fatty acids, sugar, various vitamins, essential amino acids, and water. Based on the data mining, we found that 10-hydroxy-2-decenoic acid is the principal compound and plays a crucial role in several biological processes such as inflammation and oxidative stress, estrogen-like function, antibacterial, and modulating the immune system [13]. Hence, evidence proposed that Royal jelly with various pharmacological activities is identified as an efficient supplement in therapeutic medication.

Furthermore, based on clinical evidence, physical activity can ameliorate the physical fitness features, including mobility, fatigue, and central nervous system-related outcomes white matter integrity and cognition in people with MSc. Evidence has indicated that physical activity in rodents with experimental autoimmune encephalomyelitis (EAE) significantly decreased demyelination in the lumbar spinal cord and immune cell infiltration [16]. In addition, exercise may consider a preventive therapy for developing the risk of the MSc [2]. Moreover, exercise can delay disease onset and preserve the motor neurons in the lumbar spinal cord and axons, which reduces the severity of disability [2].

Based on literature review and artificial intelligence, the association of miR-155-3p and miR-34a-p with DNAJB1, DNAJB2, FOXP1, TNFSF14, and HSPA4 manifested. On the other hand, we found that miR-155-3p and miR-34a-p have been changed in multiple sclerosis compared to healthy individuals. Hence, we proposed that Royal jelly consumption and regular exercise might modulate these biological markers in multiple sclerosis and improve conditions through neuroinflammation reduction and the regeneration of oligodendrocytes that reprogram myelin.

## Results

### Bioinformatic analysis

**Fig. 1:**
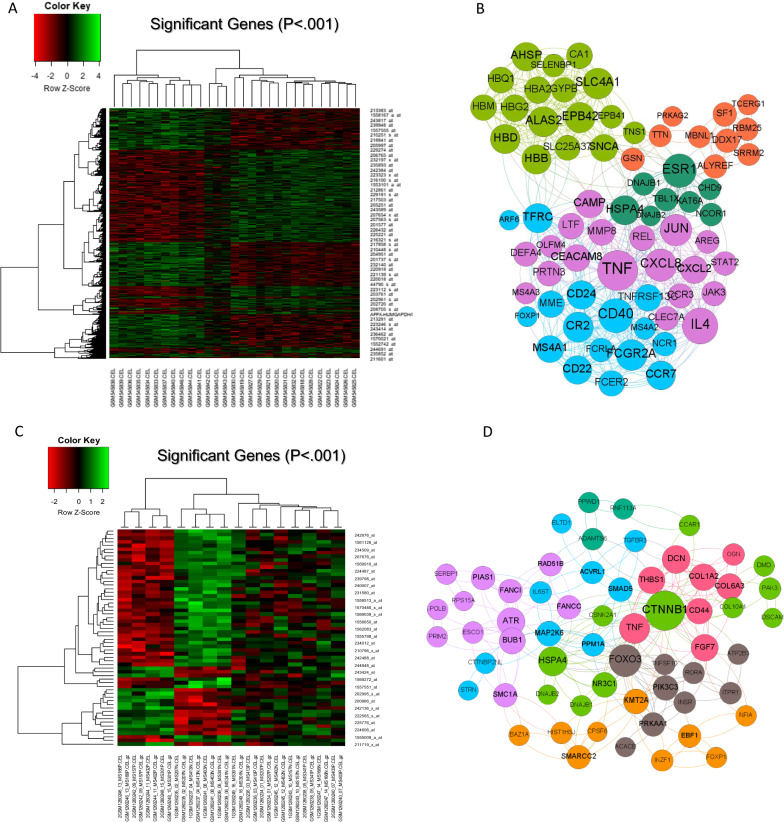
**A** The heatmap illustrates the differentially expressed genes with a *P.value* < *0.001* between patients with multiple sclerosis and healthy controls in brain-spinal samples. **B** Two hundred fifty-eight genes form a hub protein–protein interactions network associated with multiple sclerosis status in brain-spinal samples compared to healthy individuals. **C** The heatmap depicts the differentially expressed genes in blood samples from patients with multiple sclerosis and healthy controls with a *P.value* < *0.001*. **D** Compared to healthy individuals, two hundred ten genes form a hub protein–protein interactions network associated with multiple sclerosis in blood samples drawn in the STRING database

**Fig. 2:**
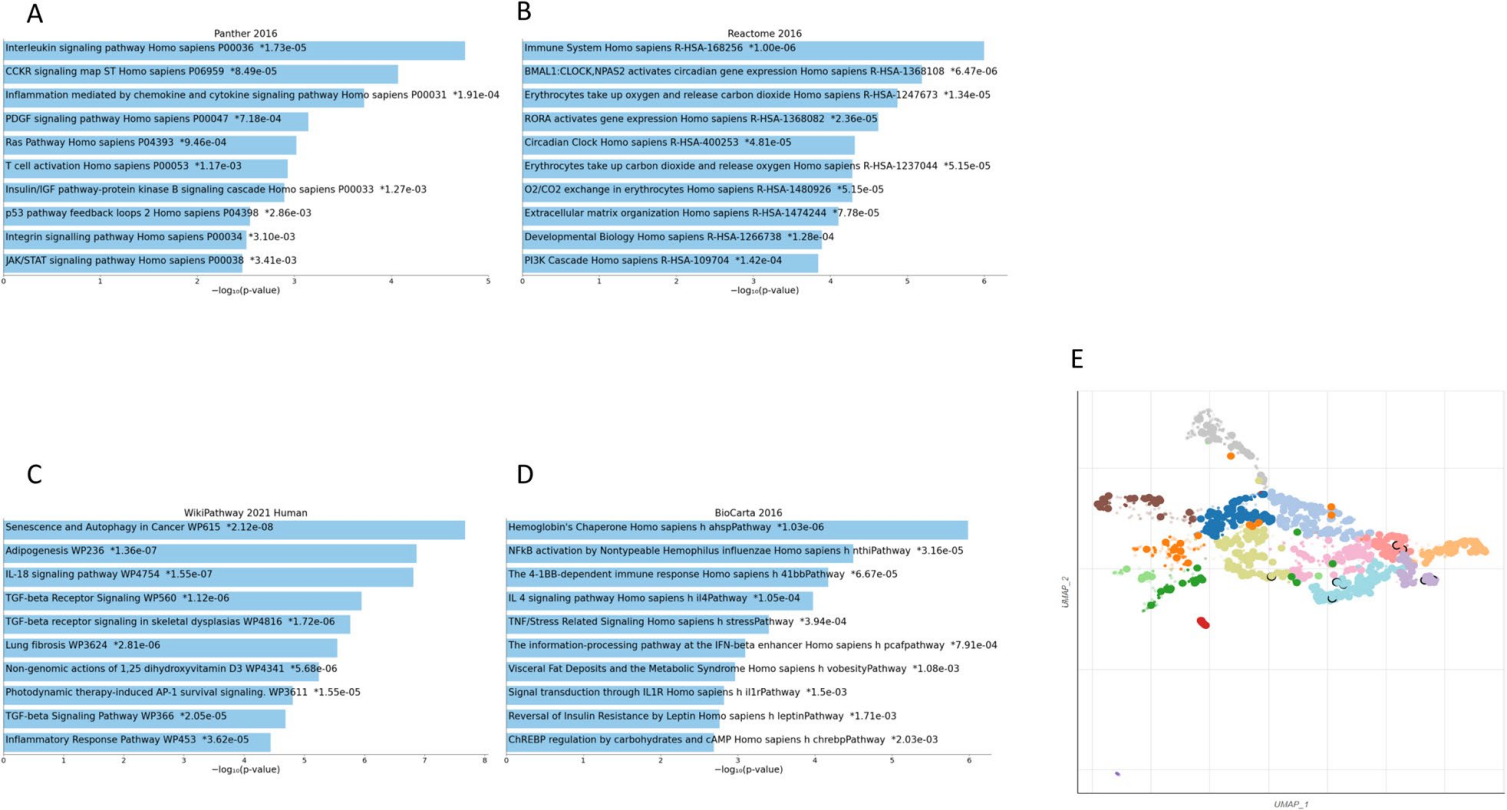
**A–E** Enrichment of hub genes involved in multiple sclerosis pathogenesis and neuron demyelination development identifies several molecular signaling pathways, including inflammation elements and pathways, extracellular matrix organization, adipogenesis, TGF-B signaling and activation, JAK/STAT signaling, and TNF/stress oxidative pathway, using KEGG, KOBAS-intelligence, and Enrich-R algorithms [55]

**Fig. 3:**
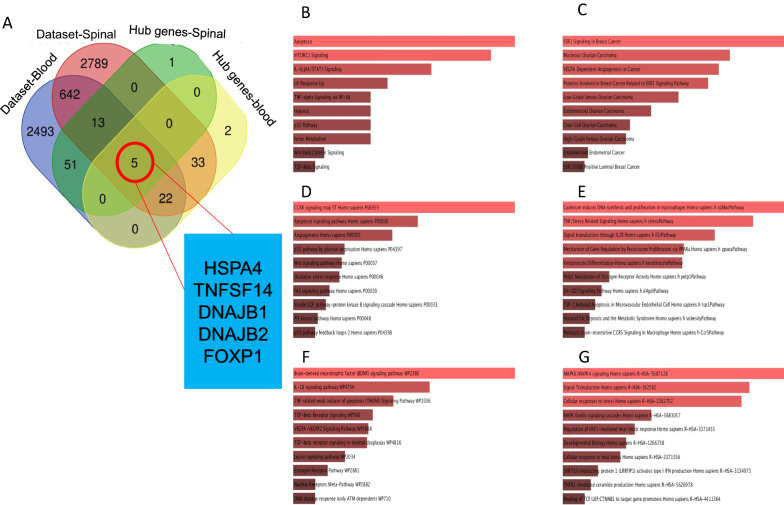
**A** The Venn diagram identified five common genes, including HSPA4, TNF, DNAJB1, DNAJB2, FOXP1 between the spinal-brain and blood datasets and the hub genes isolated from spinal-brain samples and the hub genes collected from multiple sclerosis patients’ blood samples. (**B–G**) According to common hub gene enrichment, the apoptosis and inflammation signaling pathways, hypoxia, oxidative stress, Wnt/b-catenin signaling pathway, P53 signaling pathway, TGF-B signaling pathway, and IGF/insulin signaling pathway are the most critical pathways in multiple sclerosis incidence

Based on artificial intelligence surveys and analysis of microarray dataset in spinal-brain and blood samples of multiple sclerosis patients compared to healthy individuals, we detected 379 genes with significantly differential expression in blood samples which 293 genes are overexpressed and 86 genes are downregulated. We showed differential genes expression with *P.value* < *0.001* by heatmap diagram (Fig. 1A). after applying network visualize parameters, we obtained 70 hub genes. We have drawn a protein–protein interactions network construction of 70 hub genes with associated functions in different clusters (Fig. 1B). Moreover, analysis of spinal-brain samples of the multiple sclerosis dataset highlighted 380 genes with differential expression patterns compared to healthy individuals. Among 380 genes, we identified 218 genes with negative expression patterns and 162 genes with positive expression patterns. Outcomes of gene expression pattern analysis in multiple sclerosis samples were shown in the heatmap diagram considering *P.value* < *0.001* (Fig. 1C). The protein–protein interactions network and classification in clusters based on the function of hub genes have been drawn by Gephi software after applying the network’s visualized parameters, including betweenness centrality, degree, and closeness (Fig. 1D). Enrichment of hub genes marked several molecular signaling pathways in demyelination as pathogenesis hallmark in multiple sclerosis based on browsing enrichment databases. We found that inflammation elements and pathways, extracellular matrix organization, adipogenesis, TGF-B signaling and activation, JAK/STAT signaling, and TNF/stress oxidative pathway with principal roles are involved in multiple sclerosis development. We have shown highlighted results of enrichment in Fig. 2A–E. To determine common hub genes between the spinal-brain, blood, and hub genes obtained from these datasets, we designed a Venn graph and highlighted five common genes, including DNAJB1 and DNAJB2 FOXP1, TNFSF14, and HSPA4 in these genes lists (Fig. 3A). Browsing common hub genes in several databases such as KEGG, Panther, Reactome, WikiPathway, Biocarta, and NCI Nurture marked vital signaling pathways associated with hub genes involved in multiple sclerosis pathogenesis. Apoptosis and inflammation signaling pathway, hypoxia, oxidative stress, Wnt/b-catenin signaling pathway, P53 pathway, TGF-B signaling pathway, and IGF/insulin pathway are the most critical pathways in multiple sclerosis incidence (Fig. 3B–G).

**Fig. 4:**
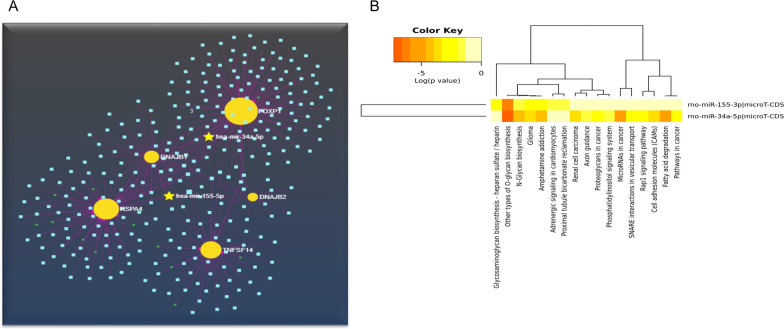
**A** The miRNet server algorithm created the network of microRNA-gene interactions. Between FOXP1 and DNAJB2, TNFSF14, and HSPA4, the miRNet algorithm identified miR-34a and miR-155 as common microRNAs. **B** Analysis and enrichment of miR-34a and miR-155-3p to determine pivotal signaling pathways related to microRNAs were marked cellular and molecular processes such as glioma, DNA damage repair, wound healing, vascular remodeling, dendritic cell differentiation, extracellular matrix remodeling, response to cytokines, inflammation, apoptosis, response to hypoxia, cell death, fatty acid degeneration, cell adhesion molecules, O-glycan and N-glycan biosynthesis, Rap-1 signaling pathway, aging processes, and pathways in cancer by Heat Map graph and miRNet algorithm enrichment

We designed a microRNAs: mRNA interaction network based on selected genes in the present study. The microRNA-155-3p interacted with DNAJB2, FOXP1, and HSPA4. On the other hand, microRNA-34a-5p was associated with FOXP1, TNFSF14, HSPA4, and DNAJB2 genes (Fig. 4A).

Analysis of microRNAs array in multiple sclerosis revealed that miR-34a-5p and miR-155-3p were up-regulated in multiple sclerosis conditions. Moreover, the prediction of microRNAs that targeted selected genes highlighted the role of miR-34a-5p and miR155-3p in multiple sclerosis pathogenesis. Enrichment of selected microRNAs based on function in the miRNet algorithm manifested that these microRNAs were associated with cellular and molecular processes such as DNA damage repair, wound healing, vascular remodeling, dendritic cell differentiation, extracellular matrix remodeling, response to cytokines, inflammation, apoptosis, response to hypoxia, cell death, and aging processes.

We designed a heatmap diagram to illustrate microRNAs-molecular and cellular signaling pathway interactions based on miRPath V.3 servers (Fig. 4B).

### Drug design results

**Fig. 5:**
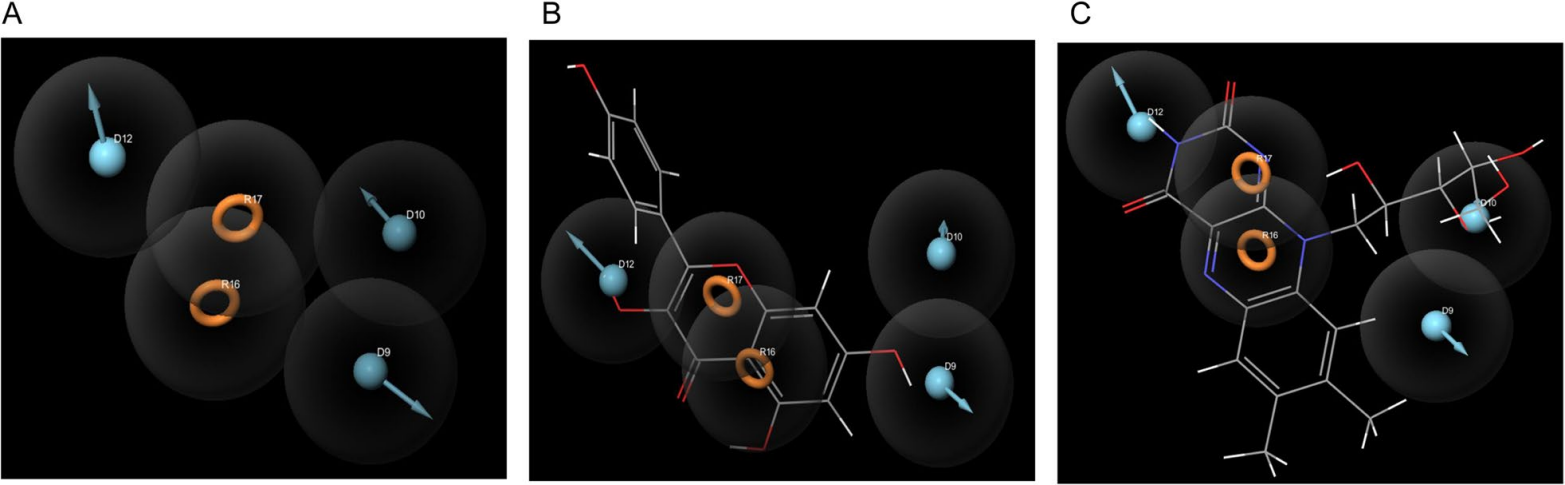
**A** The pharmacophore modeling was performed based on the active ligands library of heat-shocked proteins family 70 (HSP70) considering the suitable template (DDDRR) and the highest survival score (5.665015). (**B**, **C**) The Royal jelly bioactive compound library screening over the pharmacophore model revealed that Kaempferol (align score: 0.846716) and Vitamin B2 (align score: 0.713298) matched on pharmacophore model

Based on outcomes of computing binding affinity between bioactive compounds of Royal jelly and macromolecules involved in multiple sclerosis network with the highest degree and betweenness centrality by molecular docking practice, we concluded that all of the ten bioactive compounds of Royal jelly in this bioinformatics study bind to DNAJB1 and TNFSF14 with suitable binding affinity and acceptable RMSD threshold. Outcomes of molecular docking are presented in Table 1. Furthermore, we designed a pharmacophore model with the DDDRR template and survival score 5.665015 based on 18 active compounds documented of HSPA4 in the binding database, considering 80% of all of the library and 4 to 5 feature selection (Fig. 5A). The screening of Royal jelly’s bioactive compounds library over the pharmacophore model specified that Kaempferol (align score: 0.846716) and Vitamin B2 (align score: 0.713298) matched with pharmacophore models and could be proposed as the new active ligands for the management of disorders by targeting the HSP70 family (Fig. 5B, C).

**Table 1:** Computing binding affinity between bioactive compounds of Royal jelly and macromolecules by molecular docking method

Product	Bioactive compounds	PubChem ID	Binding affinity (Kcal/mol)		RMSD
			DNAJB1	TNFSF14	
Royal jelly	Genistein	5280961	− 6.2	− 6.4	< 2
	Kaempferol	5280863	− 6.3	− 6.4	< 2
	Formononetin	5280378	− 6.6	− 6.2	< 2
	Vitamin B2	493570	− 5.8	− 6.2	< 2
	Vitamin A	445354	− 6.1	− 6.5	< 2
	Naringenin	439246	− 6.2	− 6.4	< 2
	Isosakuranetin	160481	− 6.2	− 6.6	< 2
	24-methylenecholesterol	92113	− 6.5	− 7.3	< 2
	Hesperetin	72281	− 6.4	− 6.3	< 2
	Vitamin E	14985	− 5.7	− 6	< 2

### The characteristic and behavior of the multiple sclerosis model of Rat

**Fig. 6:**
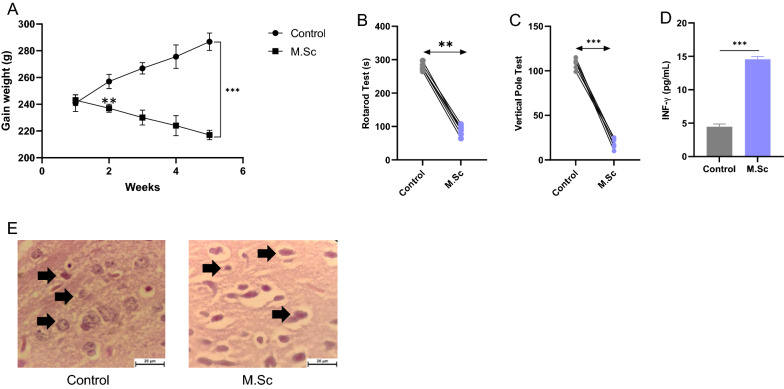
The effect of multiple sclerosis on the features, behavior, and macrophage activation factor (**A**) The gain weight of the multiple sclerosis model of rats compared with the control group. (n = 6, p < .01), (**B**, **C**) Evaluated the balance and motor coordination of the multiple sclerosis model of rats compared with the control group. (n = 6, p < .01), (**D**) The serum Interferon-gamma (IFNγ) was measured in a multiple sclerosis model of rats compared with the control group. (n = 6, p < .01), (**E**) pathological analyses were assessed in the multiple sclerosis model of rats compared with the control group with H&E staining (× 400). The dashed circle revealed the damaged cells of the nucleus. (n = 6, p < .01), ** P < .01 indicated a significant difference in multiple sclerosis rats compared with control. Data were analyzed by t-tests

The gain weight of the multiple sclerosis model of rats (M.Sc group) significantly decreased compared with the control group (Fig. 6A). Moreover, the gain weight of the M.Sc group sharply reduced in the second week after induction of multiple sclerosis Vs the Control group (Fig. 6A). In addition, this study evaluated the balance and motor coordination with the rotarod and vertical pole tests (Fig. 6B, C). The data demonstrated that the motor coordination and balance decreased in the M.Sc group compared with the control group (Fig. 6B, C). In addition, the serum concentration of the proinflammatory cytokine Interferon-gamma has indicated in Fig. 6D. In addition, the results revealed that the level of concentration of IFNγ was enhanced in the M.Sc group compared with the control group (Fig. 6D). Furthermore, in the M.Sc group, the pathological analysis indicated that the number of damaged cells of the nucleus significantly increased compared with the control group, and also, the shape of the cell was changed in the MSc group (Fig. 6E). Based on these data, the mobility, gain of weight, macrophage activation, and demyelination of the multiple sclerosis model of rats were altered compared with the control group.

### Physical activity and Royal jelly compound ameliorate the motor function, proinflammatory cytokine, and demyelination

**Fig. 7:**
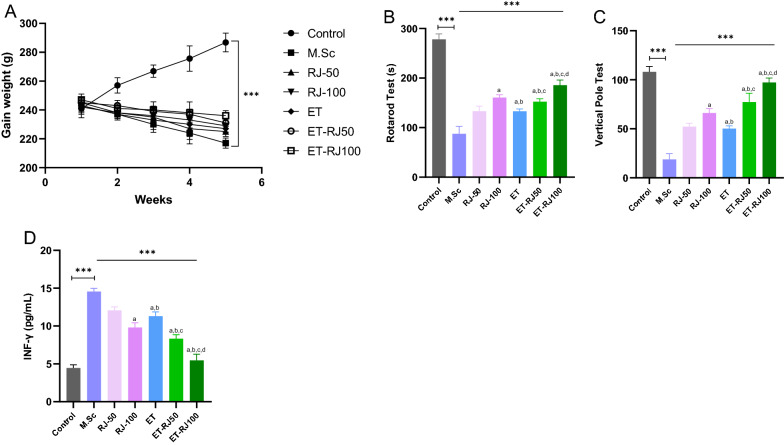
The influence of physical activity and Royal jelly on gain weight, mobility, and serum Interferon-gamma. **A** change in gain weight calculated each week. (n = 6, P < .001), (**B**, **C**) Balance and motor coordination were assessed. (n = 6, P < .001), (**D**) The serum Interferon-gamma (IFNγ) was measured in a multiple sclerosis model of rats compared with the control group. (n = 6, P < .001). One-way analysis of variance (ANOVA) and Tukey’s post hoc test was used to analyze data. a: Revealed statistically significant difference with RJ-50 group; b: Revealed statistically significant difference with RJ-100 group; c: Revealed statistically significant difference with ET group at P < 0.05; d: Revealed statistically significant difference with ET-RJ50 group

Our data indicated that the gain weight significantly was improved in multiple sclerosis rats models were treated with 50 mg/kg (RJ-50 group) and 100 mg/kg Royal jelly (RJ-100 group) in comparison M.Sc group (Fig. 7A). Moreover, physical activity (ET group) ameliorated the gain weight compared with the M.Sc group (Fig. 7A). Notably, the interaction of the exercise training and consumption of Royal jelly (ET-RJ50, ET-RJ100 groups) predominantly improved the gain weight of the multiple sclerosis rats model compared with other groups (Fig. 7A). Furthermore, data demonstrated that balance and motor coordination multiple sclerosis rat models were significantly enhanced in both 50 mg/kg (RJ-50 group) and 100 mg/kg Royal jelly (RJ-100 group) compared to the Control group. Also, Multiple sclerosis rats were treated with exercise training and 50 mg/kg Royal jelly (ET-RJ50 group), and Multiple sclerosis rats were treated with exercise training, and 100 mg/kg Royal jelly (ET-RJ100 group) were increased balance and motor coordination (Fig. 7B, C). Moreover, balance and motor coordination of sclerosis rats models were significantly increased in the ET-RJ100 group compared with other groups (Fig. 7B, C).

The macrophage activation factor and immune interferon indicated that the Interferon-gamma (IFNγ) level was reduced by consumption of the Royal jelly and physical activity. In addition, the highest reduction in the serum IFNγ was accomplished when a combination of exercise training was conducted with 100 mg/kg Royal jelly (ET-RJ100 group). As depicted, our data indicated that the concentration of the IFNγ was decreased in the RJ-50 group, RJ-100 group, and ET group compared with the M.Sc group (Fig. 7D). Notably, the level of IFNγ was not significant between the ET and RJ-100 groups. Based on these data, combining the exercise training with 100 mg/kg Royal jelly (ET-RJ100 group) effectively improved multiple sclerosis rat models.

### The effect of multiple sclerosis condition on the alternation of Dnajb1/Dnajb2/Foxp1/Tnfsf14 and Hspa4 network in the brain tissue

**Fig. 8:**
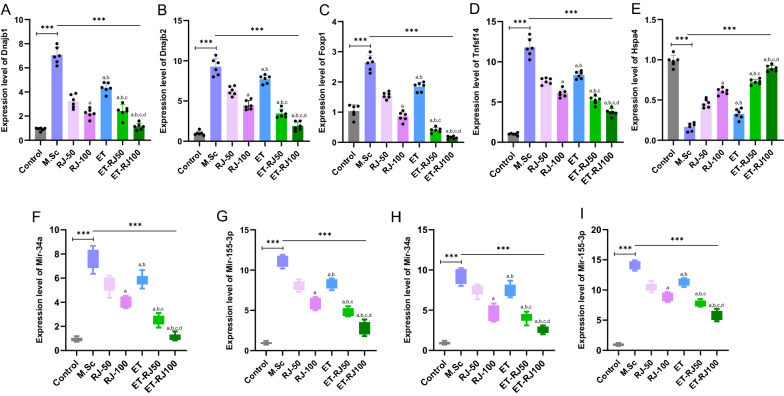
Physical activity and consumption of the Royal jelly regulated the mRNA-microRNA network in Multiple sclerosis rats. (**A–E**) the relative expression of the *Dnajb1*, *Dnajb2*, *Foxp1*, *Tnfsf14,* and *Hspa4* in the brain tissue. (**F**–**I**) the relative expression of the miR-34a-5p and miR-155-3p in the brain and blood. One-way analysis of variance (ANOVA) and Tukey’s post hoc test was used to analyze data. a: Revealed statistically significant difference with RJ-50 group; b: Revealed statistically significant difference with RJ-100 group; c: Revealed statistically significant difference with ET group at P < 0.05; d: Revealed statistically significant difference with ET-RJ50 group

Our data revealed that the relative expression of the *Dnajb1/Dnajb2/Foxp1/Tnfsf14* was increased in the M.Sc group compared with the Control group (Fig. 8A–D). Moreover, we found that the relative expression of the *Hspa4* was significantly decreased in the Multiple sclerosis rats model compared with the Control group (Fig. 8E). Based on these results and artificial intelligence analyses, the Apoptosis and inflammation signaling pathway, hypoxia, oxidative stress, Wnt/b-catenin signaling pathway, P53 pathway, TGF-B signaling pathway, IGF/insulin pathway are dysregulated in multiple sclerosis.

### Physical activity and Royal jelly compound altered the Dnajb1/Dnajb1/Foxp1/Tnfsf14 and Hspa4 network in the brain tissue

We selected a pool mRNA network based on artificial intelligence and in-silico analysis. The maximum reduction of *Dnajb1/Dnajb2/Foxp1/Tnfsf14* network was observed when Multiple sclerosis rats were treated with exercise training and 100 mg/kg Royal jelly (ET-RJ100), but the relative expression of the *Hspa4* significantly increased (Fig. 8A–E). Moreover, exercise training (ET group), 50 mg/kg Royal jelly (RJ-50), and 100 mg/kg Royal jelly (RJ-100) reduced the relative expression of the *Dnajb1/Dnajb2/Foxp1/Tnfsf14* network and increased the expression level of the *Hspa4* compared with the M.Sc group (Fig. 8A–E). Also, consumption of 100 mg/kg Royal jelly (RJ-100) significantly decreased the *Dnajb1/Dnajb1/Foxp1/Tnfsf14* network and enhanced *Hspa4* in comparison to RJ-50 and ET groups (Fig. 8A–E).

### The effect of Multiple sclerosis condition on alternation microRNAs profiles in the brain and blood

This study identified the critical microRNAs that could regulate *Dnajb1/Dnajb1/Foxp1/Tnfsf14* and *Hspa4* networks based on the *in-silico* analysis. Moreover, the relative expression of miR-34a-5p and miR155-3p was detected in blood and brain rats. Furthermore, we indicated that the expression level of the miR-34a-5p and miR155-3p significantly enhanced in the M.Sc group Vs. Control group (Fig. 8F–I). Therefore, these microRNAs profiles might consider biomarkers for prognosis and diagnosis development of Multiple Sclerosis.

### Physical activity and Royal jelly compound altered miR-34a-5p and miR155-3p in the brain and blood

Interestingly, the level expression of miR-34a-5p and miR155-3p was changed in the brain and blood by physical activity and consumption of the Royal jelly. Our data revealed that the expression level of the miR-34a-5p and miR155-3p significantly was decreased in Multiple sclerosis rats treated with exercise training and 100 mg/kg Royal jelly (ET-RJ100 group) compared with the other groups (Fig. 8F–I). Furthermore, Multiple sclerosis rats were treated with 50 mg/kg Royal jelly (RJ-50 group), 100 mg/kg Royal jelly (RJ-100 group), and exercise training (ET group) reduced the relative expression of the miR-34a-5p and miR155-3p compared with the control groups (Fig. 8F–I). Moreover, the interaction of the exercise training with 50 mg/kg Royal jelly and 100 mg/kg Royal jelly (ET-RJ50, ET-RJ100 groups) diminished the level of these microRNAs profiles, but the ET-RJ100 group compared with the ET-RJ50 group significantly decreased the relative expression of the miR-34a-5p and miR155-3p. Hence, we could conclude that the combination of the 100 mg/kg Royal jelly along with physical activity had most effective in comparison to other groups (Fig. 8F–I).

### The effect of exercise along with the Royal jelly compound on the neurons

**Fig. 9:**
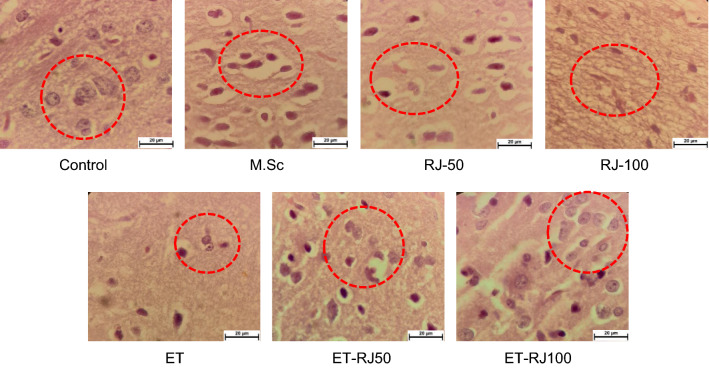
**A** The effect of the exercise and Royal jelly on damaged cells of the nucleus. Dashed circle indicated the damaged cells of the nucleus

Based on the pathological analysis, we revealed that the Royal jelly compound could reduce the number of damaged cells of the nucleus compared with the M.Sc group, and also the shape of the cell was improved in the RJ-50 group, RJ-100 group compared with the M.Sc group (Fig. 9). Moreover, we demonstrated that physical activity (ET group) could decrease the number and shape of damaged cells of the nucleus. Notably, exercise and the Royal jelly compound significantly improved the number and shape of damaged cells and reduced demyelination (Fig. 9). Furthermore, based on these data, the ET-RJ100 group predominantly diminished the demyelination of the Multiple sclerosis rats (Fig. 9).

## Discussion

The biomarkers in multiple sclerosis are defined as candidates alterable in incidence, progression, and medication reaction [17, 18]. We highlighted genes and microRNAs with the potential role in prognosis and follow-up of multiple sclerosis status based on the artificial intelligence survey.

Analysis of the closest microarray datasets of the brain, spinal cord, and blood samples of multiple sclerosis conditions manifested essential hub genes involved in the demyelination and multiple sclerosis pathogenesis. Moreover, we highlighted molecular signaling pathways associated with these hub genes. Based on enrichment and data mining, we found that inflammation elements, extracellular matrix organization, adipogenesis, TGF-B signaling and activation, JAK/STAT signaling, and TNF/stress oxidative pathway with principal roles are involved in multiple sclerosis development.

The current study revealed that all ten bioactive compounds in Royal Jelly contain Genistein, Kaempferol, Formononetin, Vitamin B2, Vitamin A, and Naringenin Isosakuranetin, 24-methylenecholesterol, Hesperetin, and Vitamin E with suitability binding affinity and RMSD. Moreover, based on the artificial intelligence analysis, we found that these bioactive compounds could bind to DNAJB1 and TNFSF14. Molecular docking computing of bioactive compounds revealed that Royal Jelly’s consumption might change critical molecular signaling pathways to ameliorate life quality and increase muscle endurance in multiple sclerosis conditions. Based on the new result, we reported that binding affinity of molecular library docking, 24-methylenecholesterol with the lowest energy (− 7.3 kcal/mol) bound to TNFSF14 and DNAJB1 proteins surface. These results led to the change of TNF pathways via Royal Jelly consumption. Furthermore, Yujuan Sun et al*.* demonstrated that the 24-methylenecholesterol bioactive compound could reverse neuron injuries and central neuron system dysfunction in multiple sclerosis. Based on these data, Yujuan Sun et al*.* suggested that 24-methylenecholesterol had anti-aging activity, anti-oxidative stress function, and neuroprotective effects [19].

Moreover, the Naringenin compound has improved Alzheimer’s disease, Type 2 Diabetes, and Cancer in several clinical trials studies [20–22]. Experiments were carried out by incorporating 5% Naringenin compound into mice’s diets or injecting 20–80 mg/kg of Naringenin compound had immunomodulatory effects and could regulate T-regs and balance the Th1/Th2 ratio in autoimmune arthritis, resulting in less inflammation [23–25]. Also, growing evidence has been shown that the Hesperetin compound had neuroprotective properties in experimental models of neurodegenerative disorders [26]. Moreover, the pharmacophore model design illustrated that the Kaempferol compound and vitamin B2 could align over the DDDRR models of the HSPA4 ligands library. Hence, vitamin B2 and Kaempferol compounds could be recommended as effective ligands for HSPA4.

This study revealed that the Royal Jell compound could improve mobility, a proinflammatory cytokine, and demyelination of Multiple sclerosis rats. Notably, among the different concentrations of Royal Jelly (50 mg/kg, 100 mg/kg), we found that 100 mg/mL had an effective and practical impact on proinflammatory cytokine and demyelination of Multiple sclerosis rats. Ample evidence has demonstrated that Royal Jelly suppressed the IL-1β, IL-18, and TNF-α levels. In addition, Royal Jelly has scavenging properties, which could reduce the antiradical and increase the anti-oxidative effect [27–29]. Interestingly, Hattori et al*.* has indicated that consumption of the Royal Jelly stimulated neuron differentiation and inhibited the production of astrocytes oligodendrocytes. In addition, Hattori found that the Royal Jelly compound had Neurotrophic effects [30, 31]. The other studies demonstrated that Royal Jelly attenuated the amyloid-beta deposition and cholesterol concentration as well as the cAMP/PKA/CREB/BDNF pathway significantly increased in mice treated with Royal Jelly. Furthermore, the autonomic nervous systems and blood–brain barrier were improved by consuming Royal Jelly [32, 33]. Moreover, we found that the relative expression of the *Dnajb1/Dnajb1/Foxp1/Tnfsf14* and *Hspa4* network was regulated by 100 mg/mL Royal compared with 50 mg/mL.

The characteristic and behavior finding showed that gain of weight significantly was improved in multiple sclerosis rats models were treated with 100 mg/kg Royal jelly (RJ-100 group) in comparison M.Sc group. This result indicated that consumption of 100 mg/kg of Royal jelly (RJ-100 group) is significantly more effective in gaining weight than 50 mg/kg (RJ-50 group). Notably, the interaction of the exercise training and consumption of Royal jelly (ET-RJ100 group) predominantly improved the gain weight of the multiple sclerosis rats model compared with other groups. Also, behavioral data displayed that balance and motor coordination in multiple sclerosis rat models were significantly enhanced in 100 mg/kg Royal jelly (RJ-100 group) compared to the Control group. Correspondingly, Multiple sclerosis rats were treated with exercise training, and 100 mg/kg Royal jelly (ET-RJ100 group) significantly increased balance and motor coordination compared with other groups. In addition, the highest reduction in the serum IFNγ was accomplished when a combination of exercise training was conducted with 100 mg/kg Royal jelly (ET-RJ100 group). Moreover, data demonstrated that 100 mg/kg of Royal jelly (ET-RJ100 group) is more efficient on expression levels of Dnajb1, Dnajb2, Foxp1, Tnfsf14, and Hspa4 in comparison with 50 mg/kg of Royal jelly (RJ-50 group). The maximum reduction of the *Dnajb1/Dnajb2/Foxp1/Tnfsf14* network was observed when Multiple sclerosis rats were treated with exercise training and 100 mg/kg Royal jelly (ET-RJ100), but the relative expression of the *Hspa4* significantly increased.

On the other hand, our data revealed that the expression level of the miR-34a-5p and miR155-3p significantly was decreased in Multiple sclerosis rats treated with exercise training and 100 mg/kg Royal jelly (ET-RJ100 group) compared with the other groups. Hence, we could conclude that the combination of the 100 mg/kg Royal jelly and physical activity had the most effective regulation in the microRNA profile compared to other groups. Based on these data, combining the exercise training with 100 mg/kg Royal jelly (ET-RJ100 group) effectively improved multiple sclerosis rat models.

Physical activity and mobility ameliorate modulating neurotransmitters in the central and peripheral nervous systems [34]. Furthermore, based on the evidence, exercise and physical activity enhanced the muscle strength and motor capacity of MS patients [35–37]. Evidence has indicated that physical activity could modulate the brain’s structure and connectome [38, 39]. Moreover, based on the literature review, exercise training might ameliorate fatigue, depression, neurogenesis, neuroprotection, remyelination, and cognition [2, 40, 41]. Lourenco and colleagues demonstrated that aerobic exercise could increase the expression level of the Nrf2 as a vital transcription factor and antioxidant enzymes activity. Based on these data, they found that the oxidative stress status was significantly reduced in the spinal cord of rodents [42]. In addition, physical activity-induced several signaling pathways in the different brain regions and neurotransmission [42]. Based on the proteomic analysis, Lozinski et al*.* revealed that the alternation of genes is related to the metabolism pathway. They indicated that the expression protein of Pgam2 in glycolysis and Ndufb9, Ndufv3, and mt-Nd3 associated with mitochondria significantly changed by exercise training [2]. In this regard, we found that physical activity improved the motor function, proinflammatory cytokine, and demyelination of the Multiple sclerosis rats. In addition, the relative expression of the *Dnajb1/Dnajb1/Foxp1/Tnfsf14* and *Hspa4* network was regulated by exercise training. Notably, we showed that the relative expression of the *Dnajb1/Dnajb1/Foxp1/Tnfsf14* was down-regulated, and the level of the *Hspa4* was up-regulated in ET compared with the M.Sc group. In addition, our data revealed that the exercise training and consumption of the Royal Jelly (ET-RJ50 and ET-RJ100) improved the *Dnajb1/Dnajb1/Foxp1/Tnfsf14* and *Hspa4* network. However, exercise training and 100 mg/kg Royal Jelly (ET-RJ100) significantly regulated the *Dnajb1/Dnajb1/Foxp1/Tnfsf14* and *Hspa4* network compared with ET-RJ50 (exercise training along with 50 mg/kg Royal Jelly). Hence, based on the characteristics and behavior (motor function, proinflammatory cytokine, and demyelination) and relative expression of the *Dnajb1/Dnajb1/Foxp1/Tnfsf14* and *Hspa4* network, we could conclude that exercise training along with 100 mg/kg Royal Jelly (ET-RJ100) had more effect on the Multiple sclerosis condition. Also, we have shown that Royal jelly with potent bioactive compounds might bind to hub genes, influence the improvement of multiple sclerosis hallmark and change the relative expression of hub genes involved in neurodegeneration to slight status. On the other hand, growing evidence revealed that physical activity could be rehabilitated processing speed in cognitive defense mechanisms, but there is no influential role in the cognitive domain [43, 44]. Our results found that synchronizing physical activity and Royal Jelly consumption could increase exercise's effect on ameliorating demyelination in multiple sclerosis hallmarks.

The microRNAs as regulators could affect gene expression in many processes. Thus, dysregulation of microRNAs and essential genes involved in the pathogenesis might develop diseases. Alteration of gene expression due to microRNAs binding to 3´UTR region of genes as a mechanism of microRNAs function is recognized that might degrade mRNA or block mRNA translation process. However, microRNAs could target the 5´UTR of coding sequences noncanonically. Here, microRNA might induce mRNA translation, and hence, upregulation of microRNA might lead to overexpressing the target gene [45–47]. In the present study, based on an *in- silico* survey, we found that miR-34a-5p and miR-155-3p binding to DNAJB2 in 5´UTR and CDS regions, respectively. Moreover, the FOXP1 targets miR-34a-5p and miR-155-3p in the CDS sequence. In addition, miR-34a-5p could target TNFSF14, and HSPA4 in CDS. On the other hand, HSPA4 is targeted by miR-155-3p in 3´UTR. Here, we revealed that the relative expression of the miR-34a-5p and miR-155-3p significantly increased in the blood and brain of Multiple sclerosis rats.

Moreover, in this study, we found the same pattern in the relative expression of these microRNA. In addition, the level expression of the miR-34a-5p and miR-155-3p were down-regulated by consumption of the Royal jelly (RJ-50, RJ-100) and exercise training (ET). Furthermore, consumption of the 100 mg/kg Royal jelly predominantly improved the level expression of the miR-34a-5p and miR-155-3p compared with the 50 mg/kg Royal jelly consumption. Marcin P. Mycko et al*.* demonstrated the upregulation of miR-155 in T-helper cells during multiple sclerosis in animal models with experimental autoimmune encephalomyelitis. Moreover, they showed that the miR-155 targeted the heat shock proteins family-40 containing Dnajb1 and Dnajb2, which could regulate Th17 cell differentiation and decrease experimental autoimmune encephalomyelitis and demyelination [9].

On the other hand, the literature review highlighted that miR-155 is a pivotal regulator in the inflammation pathway and modulated an autoimmune response. Furthermore, a recent study had shown that inhibition of miR-155 led to the prevention of pathophysiological mechanisms involved in multiple sclerosis incidence [48]. Moreover, Forough Taheri reported that miR-34 has a potential role in Th17 cell differentiation induction, and up-regulation of miR-34 could lead to progressive multiple sclerosis status, and down-regulation of miR-34 might ameliorate multiple sclerosis condition [49].

Notably, we indicated that the relative expression of the miR-34a-5p and miR-155-3p were significantly reduced by combining the exercise training and consumption of the Royal jelly. Interestingly, our data found that interaction between exercise training and 100 mg/kg Royal jelly had more effect than the other groups. One of the limitations of this study was comparing different doses of Royal jelly. We selected 50 and 100 mg/kg Royal jelly based on the data mining and literature review and found that these doses were common in previous studies [50]. In addition, after verifying our hypothesis, we suggested that consumption of the Royal jelly along with exercise training could conduct in human trials. Thus, these microRNAs could be affective biomarkers in various states of multiple sclerosis and, in addition, might be potential therapeutic candidates for the treatment of multiple sclerosis.

This study presented a list of bioactive compounds with suitable binding affinity over the DNAJB1 and TNFSF14 as the cutpoint proteins in the multiple sclerosis network based on a molecular docking survey. Nevertheless, we used total Royal Jelly as a crude drug in this study containing all of the bioactive compounds in the chemoinformatics section. Thus, we suggest that evaluating any compounds alone and/or together on multiple sclerosis should be examined in future studies to determine the mechanism and function of compounds in improving multiple sclerosis hallmarks.

## Conclusion

The artificial intelligence survey results identified hub genes and microRNAs with a potential role in diagnosing and following multiple sclerosis status. This study aimed to demonstrate that the Royal Jell compound (100 mg/kg/day) could improve mobility, proinflammatory cytokine functions, and demyelination of rats exhibiting Multiple Sclerosis-like behaviors. In addition, this study demonstrated that the combination of exercise training and 100 mg/kg Royal jelly had a more effect on regulating microRNA profiles and hub genes in rats with multiple sclerosis.

## Material and methods

### Bioinformatics analysis

We analyzed several microarray data sets obtained from the Gene Expression Omnibus database (46) in the central nervous system and blood samples in multiple sclerosis conditions to find principal hub genes involved in multiple sclerosis. Analysis of GSE52139 in spinal samples was applied by considering *P.value* < *0.05* and logarithm fold change (logFC) threshold + 1.5 highlighted 258 genes as significantly differential gene expression in multiple sclerosis. The genes with significant expression are shown in the heatmap diagram with *P.value* < *0.001*. The protein–protein interaction in this network was first drawn in the STRING server with medium confidence and based on literature evidence [51]. Then, estimating the visualizing parameters of the network such as degree = 5, betweenness centrality = 0.01, and closeness centrality = 0.10 marked 61 nodes as hub proteins in the multiple sclerosis network based on Cytoscape 3.6.0 algorithm [52].

On the other hand, we analyzed GSE21942 containing 12 blood samples of multiple sclerosis patients and 15 control subjects to identify differential gene expression in multiple sclerosis. Genes with significant expressions were collected based on *P.value* < *0.001*. Moreover, gene expression patterns were determined based on logFC threshold + 1.5, and we designed a heatmap diagram based on differential gene expression with *P.value* < *0.001*. The interaction network between 210 genes with significant differential gene expression was drawn in the STRING database [51]. Considering visualizing parameters in the protein–protein interactions (PPIs) network, including betweenness centrality = 0.01, degree = 5, and closeness centrality = 0.10, we detected 70 hub nodes involved in multiple sclerosis pathogenesis. Enrichment of hub proteins in both samples of multiple sclerosis patients highlighted molecular signaling pathways involved in demyelination pathomechanisms. The Venn diagram determined the common genes between the spinal-brain dataset, blood dataset, hup genes obtained from spinal-brain samples, and hub genes collected from multiple sclerosis blood samples patients [53]. In the next stage, we recovered the critical role of subscripted genes in signaling pathways by enrichment in several databases such as Panther, Reactome, Biocarta, WikiPathway, and KEGG [54, 55]. Finally, we selected five common hub genes with higher betweenness and the most degree among the multiple sclerosis network proteins. These genes contain HSPA4, TNFSF14, DNAJB1, DNAJB2, and FOXP1. Based on the literature review and strong evidence for diagnosis and follow-up of multiple sclerosis stages, the expression level of IFN-γ as a biomarker in multiple sclerosis was assessed in the serum of patients. Thus, in addition to five genes, we have selected measurements of IFN-γ for follow-up of multiple sclerosis patients.

Prediction of microRNAs profile with significant differential gene expression involved in multiple sclerosis highlighted 120 microRNAs with overexpressing pattern and 153 down-regulation in multiple sclerosis based on microarray dataset GSE17846 analysis (*P.value* < *0.05* and logFC + 0.2). Moreover, prediction based on target genes was conducted in miRWalk, miRNet, and miRDB servers [56]. We selected miR-34a and miR-155-3p for measurement in our in-silico survey according to prediction outcomes. The gene-microRNAs network was designed in the miRNet algorithm. The miRNet algorithm (12) network indicated that miR-34a-5p and miR-155-3p were common between FOXP1 and DNAJB2 TNFSF14 and HSPA4. An analysis of the network was conducted in the miRPath V.3 database [57] to determine the roles of selected microRNAs. Furthermore, we drew molecular signaling pathways related to highlighted microRNAs based on the Kyoto Encyclopedia of Genes and Genomes analysis (KEGG) by considering *P.value* < 0.05, microT threshold 0.4, and fisher’s exact text enrichment method.

### Drug design

Computational Molecular Biology outcomes selected five genes, including DNAJB1, DNAJB2, FOXP1, TNFSF14, and HSPA4, based on the highest visualized parameters among proteins involved in the multiple sclerosis network. The three-dimensional structure of these proteins based on X-Ray diffraction was obtained with browsed in the protein data bank (PDB) [58] with Follow-up ID 3AGZ (DNAJB1), 4J6G (TNFSF14). After preparation and optimization (removal of solvent, ligands, non-complex compounds, and sub-chains) by dock prep tool in UCSF Chimera software 1.8.1 [59], these protein structures have been prepared for molecular docking technique. To predict the effects of Royal jelly bioactive compounds on macromolecules, we required computing binding affinity and estimating binding stability between ligands and target protein. Hence, we found ten bioactive compounds in Royal jelly based on the literature review [13, 14, 60, 61]. These bioactive molecules’ three-dimensional (3D) structure was browsed in the PubChem database and obtained in SDF format [62]. We used Open Bable G.U.I. software to construct the compounds library [63]. Estimating of molecular docking between 10 compounds and macromolecules (DNAJB1 and TNFSF14) were conducted in search space with dimensions (x = 79.3563, y = 95.4487, and z = 67.6549) and (x = 47.3635, y = 66.4363, and z = 49.6427) respectively for DNAJB1 and TNFSF14 by PyRx software [64]. This molecular docking study considered the acceptable threshold for binding affinity < -5 and root-mean-square deviation of atomic positions < 2 [65].

On the other hand, we browsed reported ligands for the heat-shocked proteins family HSP70, in which HSPA4 is member 4 in this family in the Binding DataBase [66]. Pharmacophore modeling was designed based on OPLS 2005, desalting, and generating tautomers in LigPrep, considering 80% of active ligands and 4 to 5 features in PhasePharma and LigPrep applications of maestro 11.5 [67]. Based on the maximum survival score given to the pharmacophore model, we selected the suitable model and screened the alignment of 10 bioactive ligands obtained from Royal jelly on the pharmacophore model. The Royal jelly with complex and variable bioactive compounds relevant to seasonal and geographical area conditions is influenced by growth, development, reproduction, and lifespan. Although the content of compounds is variable, Royal jelly includes proteins, lipids such as free fatty acids, sugar, various vitamins, essential amino acids, and water. Based on the data mining, we found that 10-hydroxy-2-decenoic acid is the principal compound and could play a crucial role in several biological processes such as inflammation and oxidative stress, estrogen-like function, antibacterial, and modulating the immune system. Hence, evidence proposed that Royal jelly with various pharmacological activities is identified as an efficient supplement in therapeutic medication. The current study revealed that all ten bioactive compounds in Royal Jelly contain Genistein, Kaempferol, Formononetin, Vitamin B2, Vitamin A, and Naringenin Isosakuranetin, 24-methylenecholesterol, Hesperetin, and Vitamin E have an efficient supplement in therapeutic medication.

Moreover, in this chemoinformatic study, we described the binding affinity of 10 major bioactive compounds' Royal jelly over the cutpoint hub proteins, DNAJB1 and TNFSF14. We displayed that they all bind to cutpoint proteins with stable and suitable binding affinity. Since there are sufficient concentrations of any compounds in Royal Jelly, we used a cocktail of crude bioactive compounds in the experimental study.

On the other hand, it is worth noting that in this cheminformatics survey, we presented a list of bioactive compounds with suitable binding affinity over the cutpoint proteins. A list of bioactive compounds in Royal Jelly, including 17 compounds, was obtained by browsing the previous studies, that chemoinformatics computing highlighted ten bioactive compounds with the most effective molecular mechanism [68, 69].

### Ethical issue

All study procedures were approved and conducted by the Research Ethics Committees of Islamic Azad University Isfahan (Khorasgan) Branch (IR.IAU.KHUISF.REC.1400.027).

### Animal grouping and treatments

Forty-two male-Sprague Dawley rats were provided from Islamic Azad University Marvdasht Branch and were kept under standard conditions (humidity of 65% ± 5, temperature 24 ± 3 °C, and under a 12 h light–dark cycle). Rats freely accessed regular diet in a whole experimental study. We implemented myelin oligodendrocyte glycoprotein (MOG) to induce MSc in rats. For induction autoimmune encephalomyelitis (EAE) as a multiple sclerosis model, 300 mg/kg myelin oligodendrocyte glycoprotein (MOG, Sigma–Aldrich) dissolved in 100 μl phosphate-buffered salines and then were subcutaneously injected. In this study, rats were randomly divided into seven groups (n = 6):

1. The rats without treatment are called the Control group (n = 6).
2. The Rat’s induced autoimmune encephalomyelitis (EAE) as multiple sclerosis model by Intraperitoneal injection (IP) myelin oligodendrocyte glycoprotein (MOG), which is called the M.Sc group (n = 6).
3. Multiple sclerosis rats were treated with 50 mg/kg Royal jelly called the RJ-50 group (n = 6).
4. Multiple sclerosis rats were treated with 100 mg/kg Royal jelly called the RJ-100 group (n = 6).
5. Multiple sclerosis rats were treated with exercise training called the ET group (n = 6).
6. Multiple sclerosis rats were treated with exercise training and 50 mg/kg Royal jelly, the ET-RJ50 group (n = 6).
7. Multiple sclerosis rats were treated with exercise training and 100 mg/kg Royal jelly, the ET-RJ100 group (n = 6).

The Royal jelly was dissolved in normal saline and administered by gavage in two doses for five weeks (50 mg/kg for RJ-50, ET-RJ50 groups, and 100 mg/kg for RJ-100, ET-RJ100 groups) [50]. Moreover, During the experiment study, body weight was measured weekly. Finally, rats were euthanized using ketamine and xylazine. 4 ml of blood was extracted from each rat's right ventricle and placed in a sterile tube. To separate serum, blood-filled tubes were centrifuged at 1600 g for 15 min at 4 °C. Tissues and serum were collected, snap frozen, and transferred to − 80 °C for storing.

### Exercise training protocol

Rats in exercise training groups conducted physical activity on a rat-running wheel for five weeks (5 days/week). The running speed and duration exercise training protocol was set for 30 min at 11 m/min. This device could record wheel revolutions and time. Following a one-week acclimatization period on the rat-running wheel, the rotations per minute (rpm) and duration of the exercise were gradually increased (30 min at a speed of 11 m/min) [70–72]. The entire workout session lasted 30 min. The detailed intervention protocol of exercise training is provided in Table 2.

**Table 2:** Protocol of exercise training

Week	Time (min)	Rotations per minute (rpm)
1	10	5
2	15	7
3	20	9
4	25	11
5	30	11

### Protocol of royal jelly

The Royal jelly was dissolved in normal saline and administered by gavage (500 μL) in two different doses (50 mg/kg and 100 mg/kg) for five weeks (50 mg/kg for RJ-50, ET-RJ50 groups, and 100 mg/kg for RJ-100, ET-RJ100 groups). Based on the evidence and literature review, we found that royal jelly's 50 mg/kg and 100 mg/kg doses affected the immune system, redox, oxidative stress, antioxidant balance, and free radicals conditions. Moreover, according to symptoms, Multiple sclerosis is an autoimmune, acute inflammatory, and oxidative stress-mediated demyelinating disease of the central nervous system (CNS). Hence, we selected 50 mg/kg and 100 mg/kg doses to evaluate the effect of Royal jelly on MS status [50, 73–76]

### Behavior tests

1. Rotarod Test
  A Rotarod test was performed to evaluate balance and motor coordination. Rats were conducted three times per day for two days in this study. After rats were trained to acclimate to the rotarod device, the rotation speed gradually increased to 10 rpm for 30 min. The time score was calculated for each group as mean balance and motor coordination on the last day [77, 78].
2. Vertical Pole Test
  According to the previous evidence, we also evaluated the Rat’s movement disorder by Vertical Pole Test. this device included a pole (3-cm diameter) and 150-cm height. Each Rat was placed on the pole and measured the time of rats who could remind on the pole [78].

### Biochemical test

According to the manufacturer’s protocol, the serum Interferon-gamma (IFNγ) was measured by commercially available enzyme-linked immunosorbent assay ELISA Kit (Abcam, ab239425) [79].

### RNA isolation and quantitative real-time polymerase chain reaction

To confirm differential hub genes expression obtained from the bioinformatics survey, we sacrificed the multiple sclerosis Rattus models after the treatment stage with Royall jelly consumption and exercise; the brain tissues of animals were separated and snap-frozen in Liquid nitrogen and transferred to the genetics laboratory for storing in − 80 °C. In addition, the blood samples were collected, and the serum was separated for further experiments and tests. Trizol reagent (Thermo Scientific, USA) extracted the total RNA of brain tissues and blood samples based on the manufacturer’s protocol. Quantity and quality of total RNAs were verified in 260/280 nm ratio with NanoDrop spectrophotometry (Thermo Scientific, Waltham, MA, USA) and electrophoresis on the agarose gel. Treatment with DNase1 for extracted RNAs samples was performed to remove DNA contamination based on the manufacturer’s instructions (TaKaRa, Japan). For microRNA expression measurement, cDNA was synthesized by the exiqon manufacturer’s protocol without DNase1 treatment. The synthesized cDNAs were stored at − 20 °C until the next phase. Quantitative real-time PCR was performed using SYBR Green dye (TaKaRa, Kusatsu, Japan) on Rotor-Gene 6000 instrument (Corbett Life Science, Mortlake, Australia).

Sequences of primers were designed in BEACON designer and oligo seven software. The reference gene for normalization of microRNA expression is selected U6snRNA, and normalization of mRNAs expression was performed compared to the reference gene GAPDH 2^−ΔΔCt^ methods analyzed real-time PCR data.

### Pathological assessment

Immediately after the rats sacrificing, the fresh hippocamp region was separated from brain tissue. After that, the hippocamp was fixed in 10% buffered formalin for 24 h at room temperature, and then the hippocamp region was embedded in paraffin in this study. After that, fixed tissues were sliced into 5 m thick slices. Next, tissue was rehydrated and de-paraffinized with xylene, ethanol, and water based on the manuscript protocol. After that, they were stained with hematoxylin and eosin (H&E). Images were analyzed with an Olympus microscope.

### Statistical analysis

We calculated the analysis of variance with GraphPad Prism Software (Version 9 Graph Pad Software Inc., La Jolla, CA). Moreover, the Shapiro–Wilk test was analyzed for normalizing distribution. Furthermore, Data were calculated by t-test and one-way analysis of variance (ANOVA) with Tukey’s post hoc test. Data are indicated as mean ± standard deviation (SD), and differences at *P* < *0.05* demonstrated the significance in all analyses.

## Data Availability

All of the raw data and the rest of the materials are remained in Islamic Azad University Isfahan (Khorasgan) Branch and are available upon request. The datasets analysed during the current study are available in the Gene Expression Omnibus database repository, https://www.ncbi.nlm.nih.gov/geo/, https://www.ncbi.nlm.nih.gov/geo/query/acc.cgi?acc=GSE52139 PERSISTENT WEB, https://www.ncbi.nlm.nih.gov/geo/query/acc.cgi?acc=GSE21942, https://www.ncbi.nlm.nih.gov/geo/query/acc.cgi, STRING server (https://string-db.org/), Venn diagram (https://bioinfogp.cnb.csic.es/tools/venny/), http://www.pantherdb.org/https://reactome.org/, https://maayanlab.cloud/Harmonizome/dataset/Biocarta+Pathways, https://www.wikipathways.org/index.php/WikiPathways, https://www.kegg.jp/, http://mirwalk.umm.uni-heidelberg.de/, https://www.mirnet.ca/, http://mirdb.org/, http://snf-515788.vm.okeanos.grnet.gr/, https://www.rcsb.org/, https://www.rcsb.org/structure/3AGZ, https://www.rcsb.org/structure/4J6G, https://pubchem.ncbi.nlm.nih.gov/compound/Genistein, https://pubchem.ncbi.nlm.nih.gov/compound/Kaempferol, https://pubchem.ncbi.nlm.nih.gov/compound/Formononetin, https://pubchem.ncbi.nlm.nih.gov/compound/Riboflavin, https://pubchem.ncbi.nlm.nih.gov/compound/Retinol, https://pubchem.ncbi.nlm.nih.gov/compound/Naringenin, https://pubchem.ncbi.nlm.nih.gov/compound/Isosakuranetin, https://pubchem.ncbi.nlm.nih.gov/compound/24Methylenecholesterol, https://pubchem.ncbi.nlm.nih.gov/compound/Hesperetin, https://pubchem.ncbi.nlm.nih.gov/compound/Vitamin-E

## Abbreviations

qRT-PCR: Quantitative real-time PCR
ANOVA: One-way analysis of variance
MSc: Multiple sclerosis
MOG: Myelin oligodendrocyte glycoprotein
HSP40: Heat shock protein family with 40 kDa
HSP70: Heat shock protein family with 70 kDa
GWA: Genome-wide association studies
foxp1: forkhead box protein P1 loci
TNFSF: Tumor necrosis factor superfamily
logFC: logarithm fold change
PPIs: Protein–protein interactions
KEGG: Kyoto encyclopedia of genes and genomes analysis
3D: Three-dimensional
RMSD: Root-mean-square deviation
